# The development and evaluation of nine non-conventional lipid parameters for metabolic dysfunction-associated fatty liver disease in Chinese medical health examination adults: a single-center retrospective study

**DOI:** 10.3389/fnut.2026.1788704

**Published:** 2026-02-25

**Authors:** Lian Song, Lirong Zhang, Yinhui Hang, Zijie Yang, Jing Yang, Dongqing Wang

**Affiliations:** 1 Department of Medical Imaging, Affiliated Hospital of Jiangsu University, Zhenjiang, Jiangsu, China; 2 School of Medicine, Jiangsu University, Zhenjiang, Jiangsu, China

**Keywords:** ASCVD, MAFLD, non-conventional lipid parameters, screening indicators, TyG-BMI

## Abstract

**Objective:**

Metabolic dysfunction-associated fatty liver disease (MAFLD) represents a prevalent chronic hepatic condition globally, characterized by hepatic steatosis concurrent with at least one cardiometabolic risk factor, such as overweight/obesity, type 2 diabetes mellitus (T2DM), or metabolic dysregulation. This study aimed to evaluate the associations between nine non-conventional lipid parameters—BMI, NHHR, AIP, RC, GHR, CHG, LCI, TyG, TyG-BMI—and MAFLD, and to compare their predictive performance for MAFLD screening.

**Methods:**

This study utilized the electronic medical record at Wuhan Union Hospital between January 2020 and November 2021, and multi-model adjustment weighted logistic regression analysis was applied to investigate the association of the nine parameters with MAFLD. Receiver operating characteristic (ROC) curves were analyzed to assess the screening ability of the nine parameters. Furthermore, the association between the most predictive parameter and MAFLD was investigated with RCS analysis, and differences in risk across populations were explored with subgroup analyses.

**Results:**

A total of 1,592 participants were included in the final analysis, among whom 937 (58.86%) were diagnosed with MAFLD. Multivariable logistic regression identified NHHR, BMI, AIP, RC, GHR, LCI, TyG, and TyG-BMI as independent risk factors for MAFLD, with TyG-BMI demonstrating the strongest association (OR = 3.7, 95% CI: 3.05–4.48). The area under the ROC curve (AUC) for TyG-BMI was 0.81, and its predictive performance was significantly superior to that of the other parameters (all *P* < 0.001 by DeLong’s test). RCS analysis revealed a nonlinear relationship between TyG-BMI and MAFLD (*P* for nonlinearity<0.001), with an identified inflection point at a TyG-BMI value of 222.426. Additionally, MAFLD patients in the highest TyG-BMI tertile exhibited a significantly increased risk of atherosclerotic cardiovascular disease (ASCVD) compared to those in the lowest tertile (OR = 2.55, 95% CI: 1.337–4.91) after adjustment for confounders.

**Conclusion:**

The evaluated non-conventional lipid parameters, particularly TyG-BMI, are useful indicators for MAFLD identification. TyG-BMI demonstrated the strongest predictive ability for MAFLD and was independently associated with ASCVD risk in affected individuals. Elevated TyG-BMI may therefore serve as a clinically accessible marker for identifying individuals at high risk of MAFLD and for stratifying cardiovascular risk in patients with established MAFLD.

## 1 Introduction

MAFLD earlier denoted as non-alcoholic fatty liver disease (NAFLD), has become a significant worldwide health concern, and is a medical condition identified by a high level of fat within the liver, combined with at least one cardiometabolic risk factor, while excluding excessive alcohol intake and other coexisting liver conditions (1, 2). This drives a further understanding and categorizing NAFLD. MAFLD diagnosis requires imaging evidence, blood biomarkers/scoring, or histological evidence of hepatic steatosis, along with one of the following three conditions: overweight/obesity, T2DM, or abnormal metabolic disease risk (2). It has now become the leading chronic liver disease, impacting approximately one-third of adults worldwide. The prevalence of MAFLD among adults in the Asia-Pacific region ranges from 28 to 40%, with a yearly increase, it not only increases the risk of liver-related events, but also leads to extrahepatic complications--overweight/obesity, T2DM, atherosclerotic dyslipidemia, and hypertension, approximately one-third of patients may eventually develop to advanced stages, such as progressive fatty hepatitis, cirrhosis, and hepatocellular carcinoma, significantly increasing risks of liver-related and adverse cardiovascular disease (CVD) outcomes (3–6). MAFLD has increasingly become a public health concern in China. Characterized by insidious onset and progression, its severity and prevalence are frequently underestimated (7). Therefore, developing effective non-invasive screening methods and diagnostic indicators remains crucial for MAFLD prevention and control.

The exact mechanism of MAFLD remains unclear. It is widely believed that when intrahepatic adipose accumulation exceeds 5% of liver mass, metabolic disorders may occur, including abnormal fatty acid oxidation, oxidative stress, mitochondrial dysfunction, protein homeostasis disruption, and gut microbiota imbalance (8). Patients with MAFLD often exhibit dyslipidemia as high as 70%. Among patients with dyslipidemia, the prevalence of MAFLD ranges from 27 to 92%, while 69.2% of MAFLD patients also have dyslipidemia, characterized by elevated triglycerides (TG), total cholesterol (TC), low-density lipoprotein cholesterol (LDL-C) and reduced high-density lipoprotein cholesterol (HDL-C) in traditional lipid parameters, which are hallmark features of atherogenic dyslipidemia (9, 10).

However, it cannot reliably diagnose MAFLD caused by fat accumulation by relying on above these single biomarkers. Therefore, we developed and evaluated nine non-conventional lipid parameters for identifying MAFLD: (1) body mass index (BMI), (2) non-HDL-to-HDL cholesterol ratio (NHHR), (3) atherogenic index of plasma (AIP), (4) cholesterol, high-density lipoprotein, and glucose index (CHG), (5) FBG to HDL-C (GHR), (6) remnant cholesterol (RC), (7) lipoprotein combine index (LCI), (8) triglyceride-glucose index (TyG), and (9) TyG derivative indicators (TyG-BMI). These indicators are closely associated with insulin resistance (IR), obesity, T2DM, and CVD. Previous studies indicated that NHHR and AIP were more strongly associated with MAFLD risk than other traditional lipid indicators, and exhibited a significant linear positive relationship (11–13). Elevated RC and CHG levels significantly increased the incidence and mortality rates associated with CVD (14, 15). GHR and the prevalence of NAFLD was an inverted L-shaped curve relationship and saturation effect, with an inflection point of 7.443 (16). Significant nonlinear associations were observed between LCI and prediabetes risk in a Chinese population (17). TyG and TyG-BMI showed significant correlations with all-cause mortality, cardiovascular mortality, and diabetes mortality in NAFLD/Metabolic dysfunction-associated steatotic liver disease (MASLD) patients, with the association demonstrating a clear nonlinear trend (18).

Despite existing research on the association of certain non-conventional lipid parameters—including BMI, NHHR, AIP, TyG, and TyG-BMI—with MAFLD, determining the optimal predictor remains a challenge (19). In the present study, we systematically compared nine non-conventional lipid parameters against conventional parameters and conducted a comprehensive assessment of their respective predictive capacities for MAFLD risk. Our analysis aimed to identify the parameter with the highest discriminative performance, thereby offering potential clinical utility for enhancing MAFLD screening and diagnostic strategies.

## 2 Methods

### 2.1 Research design

This study performed a secondary analysis based on medical health examination data that was extracted from the electronic medical records system of Wuhan Union Hospital between January 2020 and November 2021. All details pertaining to participant enrollment have been comprehensively described in a previous publication (20). In summary, the research team from Wuhan Union Hospital recruited 1,830 people aged 40–79 years who voluntarily underwent liver ultrasound as components of a health examination. Following the exclusion of individuals with a documented history of reported excessive alcohol intake and known liver disease, diagnosed acute illness, renal insufficiency or active cancer, as well as those receiving oral or injectable steroids, and participants with incomplete biochemical measurements or missing medical history records, a total of 1,592 subjects were finally included for analysis. The diagnosis of fatty liver was established based on conventional abdominal B-mode ultrasonography conducted by trained technicians. Then, based on the diagnostic criteria of MAFLD with one or more of the following (2): (1). Overweight or obesity; (2). Diabetes; (3). All of the following items at least meet two metabolic abnormalities: (3.1). Waist circumference (WC) ≥ 102 cm in men and ≥ 88 cm in women; (3.2). Blood pressure ≥130/85 mmHg; (3.3). TG ≥ 1.70 mmol/L; (3.4). HDL-C <1.0 mmol/L for men and <1.3 mmol/L for women; (3.5). Prediabetes (i.e., fasting blood glucose (FBG) 5.6 to 6.9 mmol/L, or HbA1c 5.7 to 6.4%); (3.6). Homeostasis model assessment of insulin resistance (HOMA-IR) score ≥ 2.5; (3.7). High-sensitivity C-Reactive Protein (hs-CRP) ≥ 2 mg/L. Individuals identified with fatty liver in the absence of the aforementioned hepatic comorbidities were classified as having MAFLD. In a prior study, Yan F et al. utilized this same patient cohort to investigate the association between fat-to-muscle ratio (FMR) and NAFLD, subsequently depositing the complete anonymized dataset for all participants in the Dryad database (21). Furthermore, Yan F et al. reported that all enrolled patients were approved by the Institutional Review Board of Tongji Medical College, Huazhong University of Science and Technology (S155). As the research involved the retrospective analysis of anonymized medical records, the requirement for informed consent was waived. Clinical trial number: not applicable.

On this basis, the current study utilized the aforementioned cohort data to develop and evaluate nine non-conventional lipid parameters at medical health examination in participants with MAFLD. As a secondary analysis of anonymized pre-existing data, this investigation adhered to the ethical principles outlined in the Declaration of Helsinki.

### 2.2 Data collection and definitions

During the health examination, all participants completed a detailed questionnaire covering demographic characteristics, lifestyle factors, and medical history. The questionnaire collected self-reported information on sex, age, tobacco use and alcohol use, as well as prior medical diagnoses and current medication use. Anthropometric measurements were also taken, including body weight, height, calculated body mass index (BMI), and both diastolic (DBP) and systolic blood pressure (SBP). Laboratory assessments encompassed platelet count (PLT), TC, TG, HDL-C, LDL-C, alanine aminotransferase (ALT), aspartate aminotransferase (AST), uric acid (UA), and FBG. Liver fibrosis was assessed using the fibrosis-4 index (FIB-4) score, with cutoff values of <1.30 (low risk), 1.30–2.67 (moderate risk), and >2.67 (high risk). Given the limited number of high-risk participants (FIB-4 > 2.67), moderate and high risk categories were merged for analysis. Atherosclerotic cardiovascular disease (ASCVD) risk was stratified using a 10% threshold, with scores above this level classified as high risk and scores equal to or below 10% defined as low risk.

### 2.3 Definition of nine non-conventional lipid parameters

In this study, the nine non-conventional lipid parameters screening indicators include BMI, NHHR, AIP, RC, GHR, LCI, CHG, TyG, TyG-BMI. The formulas for their calculation are summarized as follows:

(1) BMI = Weight (kg) / [Height (m)]^2^.
(2) NHHR = (TC [mg/dL]−HDL-C [mg/dL]) / HDL-C (mg/dL).
(3) AIP = log (TG [mg/dL] / HDL-C [mg/dL]).
(4) RC = TC (mmol/L)−HDL-C (mmol/L)−LDL-C (mmol/L).
(5) GHR = FBG (mg/dL) / HDL-C (mg/dL).
(6) CHG = ln (TC[mg/dL] × FBG[mg/dL]/2 × HDL-C[mg/dL]).
(7) LCI = (TC[mmol/L] × TG [mmol/L] × LDL-C [mmol/L])/HDL-C (mmol/L).
(8) TyG = ln (TG [mg/dL] × FBG [mg/dL]/2).
(9) TyG-BMI = TyG × BMI.

Ultimately, these parameters were calculated utilizing established formulas in conjunction with the collected survey and examination data.

### 2.4 Statistical analysis

Participants were stratified into two groups according with and without MAFLD. Continuous variables are reported as mean (standard deviation) for normally distributed data and median (lower quartile, upper quartile) for non-normally distributed data. Categorical variables are summarized as frequencies (percentages). For comparisons between groups, independent samples *t*-tests were applied to normally distributed continuous variables, while the Kruskal–Wallis H test was used for non-normally distributed data. Differences in categorical variables were assessed using the chi-square test. Multivariable logistic regression models were employed to evaluate the associations between nine non-conventional lipid parameters screening indicators and MAFLD. Nine non-conventional lipid parameters screening indicators were modeled as both a continuous variable, a per 1 standard deviation (SD) increment variable and a categorical variable (tertiles). Meanwhile, we further evaluate multicollinearity and the Variance Inflation Factor (VIF) values were calculated for all covariates in the Supplementary Table 1. The inclusion of these covariates (BMI, TC, TG, LDL-C) in the same model resulted in VIF >5, suggesting strong collinearity; thus, these covariates were excluded from all fully adjusted models. FBG and HDL-C were excluded from the fully adjusted model to avoid overadjustment, as it was already a component of the MAFLD definition. So, three regression models were constructed: Model 1 was unadjusted; Model 2 adjusted for sex, age, tobacco use, alcohol use, hypertension, diabetes; Model 3 further adjusted for ALT, AST, UA, PLT, DBP, SBP. Results are presented as odds ratios (ORs) with corresponding 95% confidence intervals (CIs) and *p*-values.

ROC curves were generated and the AUC was calculated to assess the discriminative ability of each indicator. Net reclassification index (NRI) and integrated discrimination improvement (IDI) index were also employed to further assess the incremental predictive utility compared to the basic model. Sensitivity analysis was performed via 500 iterations of bootstrap resampling to evaluate the stability of the AUC estimates. The optimal cut-off value was determined by maximizing the Youden index (sensitivity + specificity – 1). After identifying the indicator with the strongest predictive performance, RCS regression was used to examine potential nonlinear relationship with MAFLD. Threshold effect analysis was conducted to detect inflection points, and a two-piecewise linear regression model was applied to estimate the location of any identified threshold. Subgroup analyses were subsequently performed to evaluate the consistency of the association between the selected predictor and MAFLD across populations stratified by sex, age, smoking use, alcohol use, hypertension, and diabetes. Results were visualized using forest plots. Interaction effects were tested, with a P-interaction >0.05 indicating stability of the association across subgroups. If significant interaction was observed, additive and multiplicative interaction analyses were performed by including product terms in the regression model. Meanwhile, we also conducted a sensitivity analysis with the diagnostic criteria of MASLD in the Supplementary material. All analyses were conducted using R version 4.2.2 (The R Foundation, Vienna, Austria). Statistical significance was defined as a two-tailed *p*-value <0.05.

## 3 Results

### 3.1 Clinical baseline characteristics

A total of 1,592 participants were included in the analysis, of whom 937 (58.86%) were diagnosed with MAFLD and 655 (41.14%) without MAFLD. The overall cohort comprised 27.9% females, and 66.8% were under 60 years of age. Compared to the non-MAFLD group, participants with MAFLD exhibited a higher proportion of males, smokers, and drinkers, and a greater percentage of individuals below 60 years old. Furthermore, significant differences (*P*<0.05) were observed between the two groups regarding the prevalence of hypertension and diabetes, as well as in laboratory and clinical measures including ALT, AST, UA, FBG, TC, TG, HDL-C, SBP, DBP, FIB-4 score, ASCVD risk scores, and eight non-conventional lipid parameters. A detailed summary of baseline characteristics for both groups were provided in Table 1 and Supplementary Table 2.

**Table 1:** Baseline characteristics of individuals with and without MAFLD.

Variables	Total (*n* = 1,592)	Non-MAFLD (*n* = 655)	MAFLD (*n* = 937)	*p* value
Sex, *n* (%)				<0.001
Female	444 (27.9)	247 (37.7)	197 (21)	
Male	1,148 (72.1)	408 (62.3)	740 (79)	
Age (years), *n* (%)				0.121
<60	1,063 (66.8)	423 (64.6)	640 (68.3)	
≥60	529 (33.2)	232 (35.4)	297 (31.7)	
PLT (10^9^ /L)	211.1 ± 53.3	209.8 ± 52.7	212.1 ± 53.8	0.41
ALT (U/L)	26.2 ± 19.8	20.6 ± 13.6	30.2 ± 22.3	<0.001
AST (U/L)	23.1 ± 11.0	21.4 ± 9.6	24.3 ± 11.7	<0.001
UA (umol/L)	366.8 ± 95.8	336.4 ± 85.9	388.0 ± 96.7	<0.001
FBG (mmol/L)	5.5 ± 1.7	5.2 ± 1.4	5.8 ± 1.8	<0.001
TC (mmol/L)	4.5 ± 1.1	4.4 ± 1.0	4.5 ± 1.1	0.032
TG (mmol/L)	1.9 ± 1.7	1.4 ± 1.1	2.3 ± 1.9	<0.001
HDL-C (mmol/L)	1.1 ± 0.3	1.2 ± 0.4	1.0 ± 0.3	<0.001
LDL-C (mmol/L)	2.7 ± 0.9	2.7 ± 0.9	2.7 ± 0.9	0.817
SBP (mm Hg)	130.9 ± 15.8	128.6 ± 15.5	132.6 ± 15.9	<0.001
DBP (mm Hg)	81.6 ± 11.2	79.5 ± 11.0	83.1 ± 11.1	<0.001
Tobacco use, *n* (%)				<0.001
No	1,054 (66.2)	483 (73.7)	571 (60.9)	
Yes	538 (33.8)	172 (26.3)	366 (39.1)	
Alcohol use, *n* (%)				<0.001
No	1,072 (67.3)	484 (73.9)	588 (62.8)	
Yes	520 (32.7)	171 (26.1)	349 (37.2)	
Hypertension, *n* (%)				<0.001
No	649 (40.8)	345 (52.7)	304 (32.4)	
Yes	943 (59.2)	310 (47.3)	633 (67.6)	
Diabetes, *n* (%)				<0.001
No	1,094 (68.7)	529 (80.8)	565 (60.3)	
Yes	498 (31.3)	126 (19.2)	372 (39.7)	
BMI (kg/m^2^)	25.4 ± 2.9	23.9 ± 2.4	26.5 ± 2.8	<0.001
TyG	8.8 ± 0.7	8.5 ± 0.6	9.0 ± 0.7	<0.001
AIP	0.5 ± 0.3	0.4 ± 0.3	0.6 ± 0.3	<0.001
GHR	2.5 ± 1.1	2.2 ± 1.0	2.8 ± 1.1	<0.001
TyG-BMI	223.8 ± 36.3	202.2 ± 27.3	238.9 ± 34.2	<0.001
CHG	13.4 ± 0.5	13.4 ± 0.5	13.4 ± 0.5	0.511
LCI	25.5 ± 30.6	16.8 ± 19.8	31.7 ± 35.0	<0.001
NHHR, M (P_25_, P_75_)	3.1 (2.2, 4.0)	2.7 (2.0, 3.5)	3.4 (2.5, 4.4)	<0.001
RC, M (P_25_, P_75_)	0.6 (0.4, 0.8)	0.5 (0.3, 0.7)	0.7 (0.4, 0.9)	<0.001
FIB-4 score, M (P_25_, P_75_)	0.8 (0.0, 1.3)	0.0 (0.0, 0.0)	1.2 (0.9, 1.6)	<0.001
ASCVD risk score, M (P_25_, P_75_)	9.7 (4.2, 18.6)	7.7 (2.6, 16.6)	11.0 (5.4, 20.3)	<0.001

PLT, platelets; ALT, alanine aminotransferase; AST, aspartate aminotransferase; UA, uric acid; FBG, fasting blood glucose; TC, total cholesterol; TG, triglycerides; HDL-C, high-density lipoprotein cholesterol; LDL-C, low-density lipoprotein cholesterol; SBP, systolic blood pressure; DBP, diastolic blood pressure; BMI, body mass index; TyG, triglyceride-glucose index; AIP, atherogenic Index of Plasma; GHR, fasting blood glucose to HDL-C; LCI, lipoprotein combine index; RC, remnant cholesterol; NHHR, non-HDL-to-HDL cholesterol ratio; CHG, cholesterol, high-density lipoprotein, and glucose index; M (P_25_, P_75_), median (lower quartile, upper quartile); FIB-4 score, fibrosis-4 score; ASCVD, atherosclerotic cardiovascular disease; MAFLD, metabolic dysfunction-associated fatty liver disease.

### 3.2 Association between nine non-conventional lipid parameters and MAFLD

Multivariable logistic regression analysis demonstrated that eight of the nine evaluated non-conventional lipid parameters exhibited a significant association with MAFLD across unadjusted and partially adjusted models, as presented in Table 2 and Supplementary Table 3. Each parameter was analyzed as a continuous variable, a per 1-SD increment, in tertiles, and for trend. Eight parameters showed significant associations with MAFLD risk in Model 1 and Model 2. However, in the fully adjusted Model 3, the RC showed a significant association in the highest tertile when analyzed as a categorical variable and for trend. The CHG showed significant association only at the highest tertile of Model 1 and in trend analysis. Whether evaluated as a per 1-SD increment variable or categorical variables, TyG-BMI displayed the strongest association with MAFLD, with OR of 3.7 (95% CI, 3.05–4.48) and 9.87 (95% CI, 6.99–13.94). This was followed, in descending order of association magnitude, by BMI (OR = 2.9, 95% CI: 2.44–3.45), TyG (OR = 1.99, 95% CI: 1.71–2.32), AIP (OR = 1.85, 95% CI: 1.6–2.14), RC (OR = 1.69, 95% CI: 1.39–2.06), LCI (OR = 1.66, 95% CI: 1.37–61.99), NHHR (OR = 1.47, 95% CI: 1.27–1.68), GHR (OR = 1.47, 95% CI: 1.25–1.73), and CHG (OR = 1.06, 95% CI: 0.94–1.19).

**Table 2:** Relationship between nine non-conventional lipid parameters and MAFLD in different models.

Variables	Model 1	*p* value	Model 2	*p* value	Model 3	*p* value
	OR (95% CI)		OR (95% CI)		OR (95% CI)	
NHHR	1.55 (1.42–1.69)	<0.001	1.51 (1.37–1.65)	<0.001	1.31 (1.19–1.45)	<0.001
Per 1-SD increment	1.85 (1.64–2.08)	<0.001	1.78 (1.56–2.03)	<0.001	1.47 (1.27–1.68)	<0.001
NHHR (tertiles)						
Q1	Reference		Reference		Reference	
Q2	1.86 (1.46–2.37)	<0.001	1.91 (1.47–2.47)	<0.001	1.63 (1.24–2.14)	<0.001
Q3	3.63 (2.81–4.71)	<0.001	3.38 (2.55–4.46)	<0.001	2.24 (1.66–3.02)	<0.001
P for trend	<0.001		<0.001		<0.001	
BMI	1.55 (1.47–1.63)	<0.001	1.5 (1.41–1.58)	<0.001	1.44 (1.36–1.53)	<0.001
Per 1-SD increment	3.58 (3.06–4.19)	<0.001	3.24 (2.74–3.83)	<0.001	2.9 (2.44–3.45)	<0.001
BMI (tertiles)						
Q1	Reference		Reference		Reference	
Q2	4.63 (3.71–5.79)	<0.001	3.89 (3.08–4.92)	<0.001	3.46 (2.71–4.41)	<0.001
Q3	13.39 (7.05–25.42)	<0.001	8.6 (4.46–16.59)	<0.001	6.33 (3.24–12.38)	<0.001
P for trend	<0.001		<0.001		<0.001	
TyG	4.02 (3.31–4.88)	<0.001	3.35 (2.74–4.11)	<0.001	2.6 (2.1–3.21)	<0.001
Per 1-SD increment	2.72 (2.37–3.13)	<0.001	2.39 (2.07–2.77)	<0.001	1.99 (1.71–2.32)	<0.001
TyG (tertiles)						
Q1	Reference		Reference		Reference	
Q2	2.41 (1.88–3.09)	<0.001	3.18 (2.45–4.13)	<0.001	2.67 (2.03–3.5)	<0.001
Q3	7.87 (5.93–10.45)	<0.001	6.85 (5.07–9.24)	<0.001	4.63 (3.37–6.36)	<0.001
P for trend	<0.001		<0.001		<0.001	
AIP	14.89 (10.18–21.79)	<0.001	10.54 (7.05–15.76)	<0.001	6 (3.93–9.17)	<0.001
Per 1-SD increment	2.52 (2.21–2.87)	<0.001	2.24 (1.95–2.57)	<0.001	1.85 (1.6–2.14)	<0.001
AIP (tertiles)						
Q1	Reference		Reference		Reference	
Q2	2.37 (1.85–3.03)	<0.001	2.2 (1.7–2.85)	<0.001	1.76 (1.34–2.31)	<0.001
Q3	7 (5.27–9.28)	<0.001	6.19 (4.58–8.35)	<0.001	4.08 (2.97–5.61)	<0.001
P for trend	<0.001		<0.001		<0.001	
						
RC	4.21 (3.07–5.77)	<0.001	3.25 (2.38–4.46)	<0.001	2.29 (1.68–3.11)	<0.001
Per 1-SD increment	2.49 (2.04–3.05)	<0.001	2.12 (1.73–2.59)	<0.001	1.69 (1.39–2.06)	<0.001
RC (tertiles)						
Q1	Reference		Reference		Reference	
Q2	1.69 (1.32–2.15)	<0.001	1.46 (1.13–1.89)	0.004	1.3 (1–1.7)	0.054
Q3	4.06 (3.13–5.28)	<0.001	3.24 (2.46–4.28)	<0.001	2.31 (1.72–3.1)	<0.001
P for trend	<0.001		<0.001		<0.001	
GHR	2.06 (1.8–2.36)	<0.001	1.66 (1.43–1.93)	<0.001	1.42 (1.22–1.65)	<0.001
Per 1-SD increment	2.22 (1.91–2.58)	<0.001	1.75 (1.48–2.07)	<0.001	1.47 (1.25–1.73)	<0.001
GHR (tertiles)						
Q1	Reference		Reference		Reference	
Q2	2.8 (2.18–3.59)	<0.001	2.38 (1.83–3.09)	<0.001	1.83 (1.39–2.41)	<0.001
Q3	4.7 (3.62–6.11)	<0.001	2.96 (2.2–3.99)	<0.001	2.08 (1.52–2.85)	<0.001
P for trend	<0.001		<0.001		<0.001	
TyG-BMI	1.04 (1.04–1.05)	<0.001	1.04 (1.04–1.05)	<0.001	1.04 (1.03–1.04)	<0.001
Per 1-SD increment	4.85 (4.07–5.79)	<0.001	4.33 (3.6–5.21)	<0.001	3.7 (3.05–4.48)	<0.001
TyG-BMI (tertiles)						
Q1	Reference		Reference		Reference	
Q2	4.17 (3.22–5.41)	<0.001	3.64 (2.79–4.75)	<0.001	3.14 (2.39–4.13)	<0.001
Q3	18.09 (13.15–24.89)	<0.001	13.5 (9.68–18.82)	<0.001	9.87 (6.99–13.94)	<0.001
P for trend	<0.001		<0.001		<0.001	
LCI	1.03 (1.02–1.03)	<0.001	1.03 (1.02–1.03)	<0.001	1.02 (1.01–1.03)	<0.001
Per 1-SD increment	2.31 (1.93–2.77)	<0.001	2.16 (1.79–2.61)	<0.001	1.66 (1.37–1.99)	<0.001
LCI (tertiles)						
Q1	Reference		Reference		Reference	
Q2	1.95 (1.52–2.48)	<0.001	2.12 (1.63–2.75)	<0.001	1.8 (1.37–2.37)	<0.001
Q3	4.65 (3.57–6.07)	<0.001	4.48 (3.36–5.96)	<0.001	2.96 (2.18–4.03)	<0.001
P for trend	<0.001		<0.001		<0.001	
CHG	0.93 (0.75–1.16)	0.511	1.09 (0.85–1.4)	0.477	1.13 (0.87–1.47)	0.353
Per 1-SD increment	0.97 (0.88–1.07)	0.511	1.04 (0.93–1.17)	0.477	1.06 (0.94–1.19)	0.353
CHG (tertiles)						
Q1	Reference		Reference		Reference	
Q2	0.89 (0.7–1.14)	0.366	1.08 (0.83–1.41)	0.55	1.1 (0.84–1.45)	0.486
Q3	0.78 (0.61–1)	0.046	0.94 (0.72–1.24)	0.678	0.96 (0.72–1.29)	0.807
P for trend	<0.001		<0.001		0.822	

Model 1: unadjusted.

Model 2: sex, age, tobacco use, alcohol use, hypertension, diabetes.

Model 3: model 2 + ALT, AST, UA, PLT, SBP, DBP.

OR, odds ratio; CI, confidence interval.

### 3.3 Predictive value of nine parameters for MAFLD

**Figure 1:**
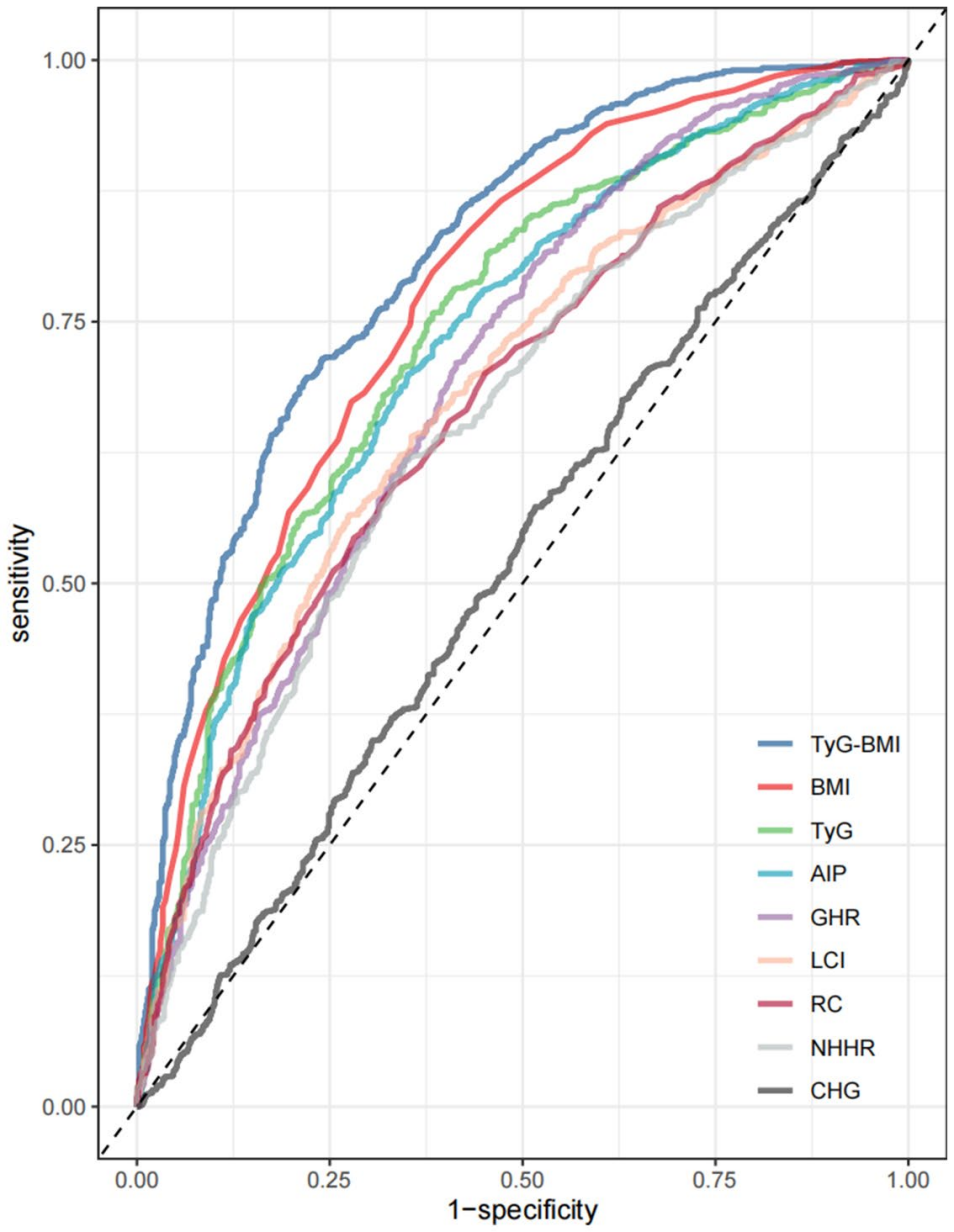
ROC curves of ten non-conventional lipid parameters for MAFLD screening in Chinese adults. ROC, receiver operating characteristic.

The AUC values and 95% CI for the nine parameters in screening for MAFLD among Chinese adults are presented in Figure 1. Among these, TyG-BMI demonstrated the highest discriminative ability for MAFLD (AUC = 0.81), followed by BMI (AUC = 0.77) and TyG (AUC = 0.74), respectively in Table 3. Consistent with these findings, Table 4 quantifies the incremental utility using NRI and IDI values. Specifically, TyG-BMI significantly improved reclassification: NRI = 0.693 (95% CI 0.6–0.786) and IDI = 0.123 (95% CI 0.107–0.139). Pairwise comparison via DeLong’s test revealed that the AUC of TyG-BMI was significantly greater than that of all other non-conventional lipid parameters (*P*<0.001). Sensitivity analysis using bootstrap resampling confirmed the robustness of the AUC values.

**Table 3:** Results of ROC analysis of nine screening tools.

Test	Cut-off value	Sensitivity	Specificity	AUC (95% CI)	*p* value (adjusted)
TyG-BMI	218.67	0.7	0.78	0.81 (0.79–0.83)	Reference
BMI	24.2	0.8	0.62	0.77 (0.75–0.8)	<0.001
TyG	8.51	0.76	0.62	0.74 (0.72–0.77)	<0.001
AIP	0.44	0.7	0.65	0.73 (0.7–0.75)	<0.001
GHR	2.12	0.71	0.59	0.7 (0.67–0.72)	<0.001
LCI	18.57	0.56	0.73	0.68 (0.66–0.71)	<0.001
RC	0.61	0.54	0.72	0.67 (0.65–0.7)	<0.001
NHHR	3.06	0.62	0.65	0.66 (0.63–0.68)	<0.001
CHG	13.47	0.57	0.48	0.52 (0.49–0.55)	<0.001

**Table 4:** Incremental predictive value of nine non-conventional lipid parameters for MAFLD.

Model	NRI (95% CI)	*p* value	IDI (95% CI)	*p* value
Basic model	Reference	Reference	Reference	Reference
TyG-BMI	0.693 (0.6–0.786)	<0.001	0.123 (0.107–0.139)	<0.001
BMI	0.626 (0.532–0.719)	<0.001	0.098 (0.083–0.112)	<0.001
TyG	0.473 (0.376–0.569)	<0.001	0.047 (0.037–0.058)	<0.001
AIP	0.436 (0.34–0.533)	<0.001	0.041 (0.031–0.051)	<0.001
GHR	0.235 (0.137–0.333)	<0.001	0.013 (0.007–0.018)	<0.001
LCI	0.272 (0.177–0.367)	<0.001	0.018 (0.012–0.025)	<0.001
RC	0.282 (0.186–0.377)	<0.001	0.018 (0.012–0.025)	<0.001
NHHR	0.26 (0.161–0.358)	<0.001	0.016 (0.01–0.022)	<0.001
CHG	0.049 (−0.051–0.149)	0.339	0.0003 (−0.001–0.001)	0.605

The basic model included sex, age, tobacco use, alcohol use, hypertension, diabetes, ALT, AST, UA, PLT, SBP, DBP.

NRl, net reclassification improvement; IDI, integrated discrimination improvement; CI, confidence interval.

### 3.4 Exploring the exposure-risk relationship between TyG-BMI and MAFLD

**Figure 2:**
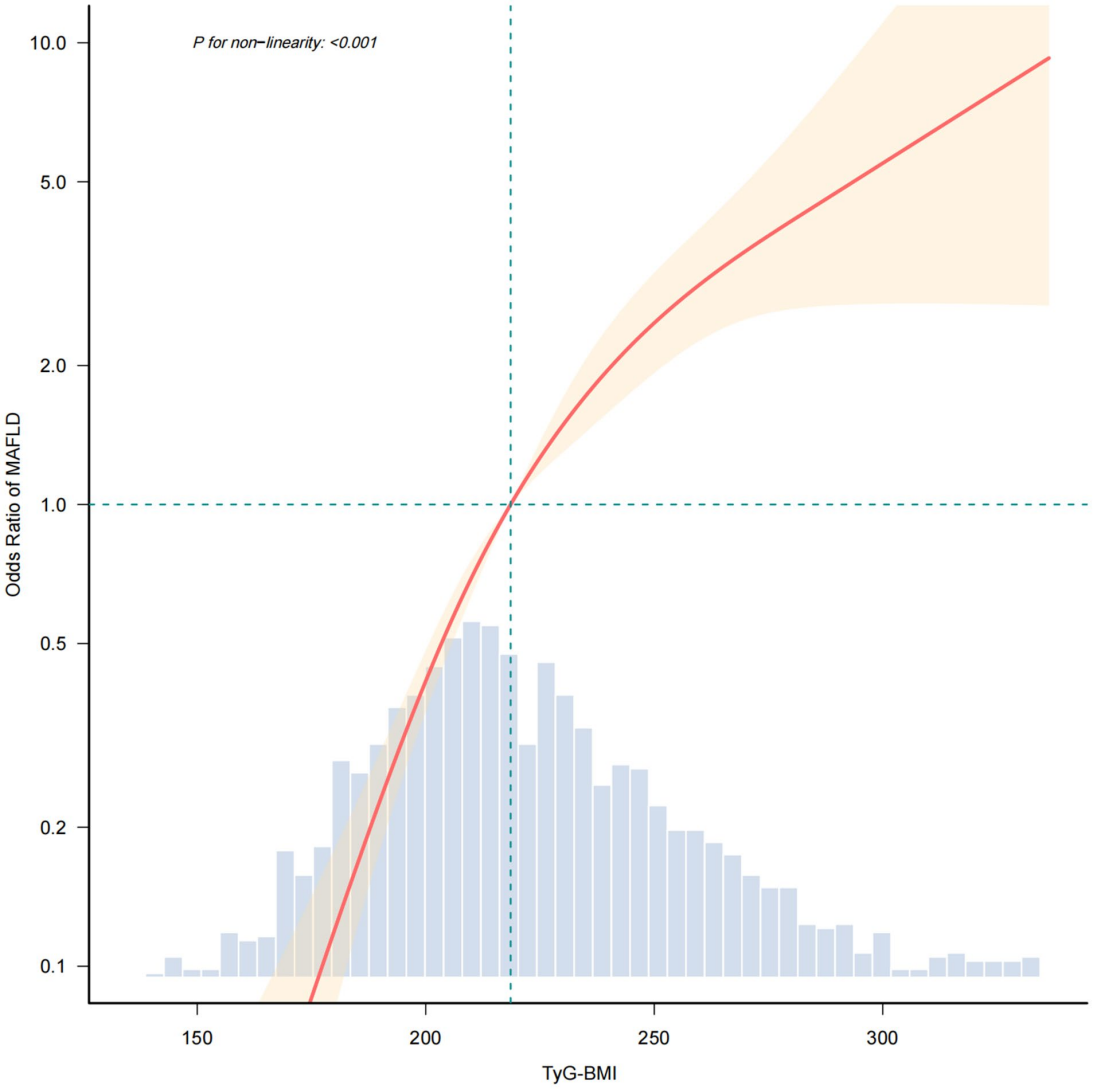
The RCS analysis of the association between TyG-BMI and MAFLD. RCS, restricted cubic spline.

Based on the preceding analysis, TyG-BMI was identified as the strongest predictor of MAFLD. To investigate the nature of this association, RCS model was applied, revealing a nonlinear relationship (*P* for nonlinearity <0.001) after adjusting for all confounders variables in Model 3 (Figure 2; Supplementary Figure 1). A segmented relationship was observed, with an inflection point at a TyG-BMI value of 222.426. Both below and above this threshold, TyG-BMI maintained a significant positive association with MAFLD (OR: 1.052, 95% CI: 1.04–1.064 and OR: 1.02, 95% CI: 1.009–1.031, respectively) (Supplementary Table 4).

**Figure 3:**
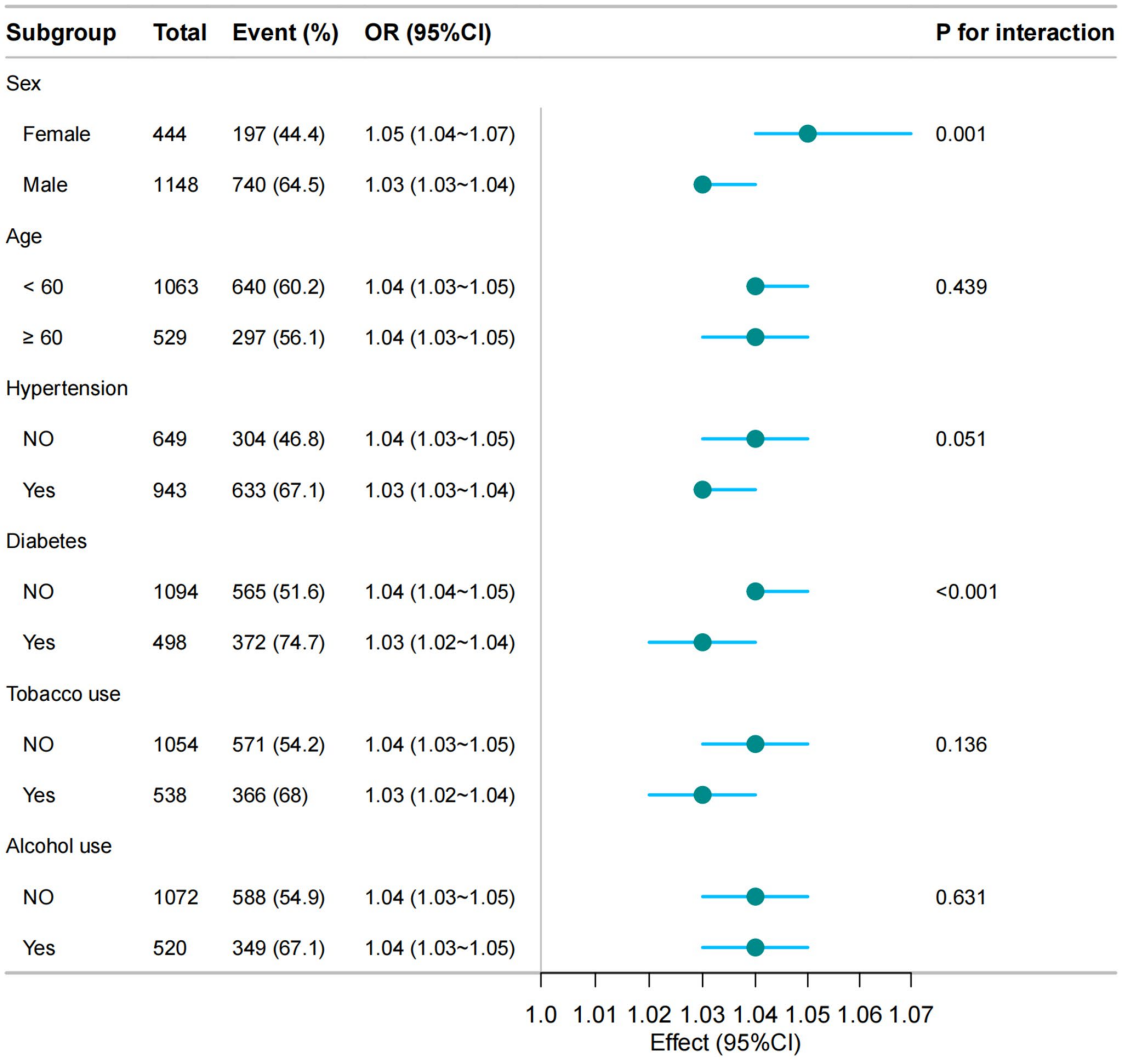
Subgroup analyses of the association between TyG-BMI and MAFLD. The model was adjusted based on Model 3.

Subgroup analyses were conducted to assess the consistency of the TyG-BMI and MAFLD association across demographic variables. As shown in Figure 3, no significant interaction was detected between TyG-BMI and the stratified variables, including age, hypertension, alcohol use, or tobacco use. Further interaction analyses evaluated potential additive and multiplicative effects between TyG-BMI and sex as well as diabetes on MAFLD risk (Supplementary Table 5). After full covariate adjustment, neither sex nor diabetes exhibited significant interaction with TyG-BMI on either scale, as indicated by RERI and AP 95% CIs spanning zero, and SI and multiplicative interaction terms encompassing one.

Given known sex-based differences in body composition, ROC analyses were performed separately for men and women, and for individuals with and without diabetes. The optimal TyG-BMI cutoff was higher in men (219.31) than in women (202.043), while the AUC was greater in women (0.86, 95% CI: 0.83–0.89) than in men (0.78, 95% CI: 0.75–0.81). Similarly, the cutoff was higher in participants with diabetes (221.19) than in those without (216.98), with a higher AUC observed in the non-diabetic group (0.83, 95% CI: 0.8–0.85) compared to the diabetic group (0.74, 95% CI: 0.69–0.79). All between-group differences in AUC were statistically significant (*P*<0.001). Corresponding sensitivity, specificity, positive predictive value, and negative predictive value are provided in Supplementary Table 6.

### 3.5 Association of TyG-BMI with risk of ASCVD and liver fibrosis in participants with MAFLD

Table 5 and Supplementary Table 7 present the association between TyG-BMI tertiles and high ASCVD risk in participants with MAFLD. Using the first tertile as reference, multivariable logistic regression analysis—adjusted for the same confounders as in Model 3—revealed that the highest tertile (OR = 2.55, 95% CI 1.33–4.91) was significantly associated with elevated ASCVD risk, whereas the second tertile showed no significant association. There was no significant association between TyG-BMI and FIB-4 in participants with MAFLD (*P* > 0.05).

**Table 5:** Association between TyG-BMI and the risk of ASCVD and liver fibrosis in patients with MAFLD.

Outcome variables	Variables	Model 1	*p* value	Model 2	*p* value	Model 3	*p* value
		OR (95% CI)		OR (95% CI)		OR (95% CI)	
ASCVD	TyG-BMI	1.01 (1.01–1.01)	<0.001	1.01 (1–1.01)	0.016	1.01 (1–1.01)	0.087
	TyG-BMI (tertiles)						
	Q1	Reference		Reference		Reference	
	Q2	1.35 (0.91–2.01)	0.13	1.97 (1.04–3.73)	0.039	1.89 (0.97–3.69)	0.063
	Q3	2.31 (1.58–3.36)	<0.001	3.01 (1.62–5.59)	<0.001	2.55 (1.33–4.91)	0.005
	P for trend	<0.001		<0.001		0.006	
Liver fibrosis risk	TyG-BMI	1 (0.99–1)	0.342	1 (1–1.01)	0.503	1 (0.99–1.01)	0.766
	TyG-BMI (tertiles)						
	Q1	Reference		Reference		Reference	
	Q2	1.05 (0.71–1.56)	0.805	1.13 (0.72–1.79)	0.592	1.73 (0.75–3.99)	0.197
	Q3	0.98 (0.67–1.43)	0.912	1.23 (0.79–1.92)	0.361	1.8 (0.78–4.14)	0.168
	P for trend	0.792		0.356		0.224	

Model 1: unadjusted.

Model 2: sex, age, tobacco use, alcohol use, hypertension, diabetes.

Model 3: model 2 + ALT, AST, UA, PLT, SBP, DBP.

OR, odds ratio; CI, confidence interval; ASCVD, atherosclerotic cardiovascular disease.

## 4 Discussion

In recent years, Asian populations exhibit higher MAFLD prevalence and shown a global upward trend. A national research report from China showed that the overall prevalence of MAFLD was 37%, with males (46%) higher than females (24%), and the prevalence gradually increases with age, which may be attributed to excessive carbohydrate intake, central obesity, and genetic susceptibility (22). The MAFLD prevalence of 58.86% observed in our cohort exceeds estimates from general population surveys in China. We attribute this discrepancy primarily to selection bias inherent to hospital-based health examination populations, who predominantly comprise white-collar workers with high socioeconomic status, sedentary occupations, and higher health-seeking behaviors—characteristics clustering MAFLD risk factors. This phenomenon has been consistently reported in tertiary center-based studies. Ma X et al. found that the proportion of individuals diagnosed with MAFLD was 50.16% among the health examination population in the Kailuan Group from July 2006 to December 2007 (*n* = 49,518) (23). Charatcharoenwitthaya P et al. also found that the proportion of individuals diagnosed with MAFLD was 45.32% among the National Health Examination Survey in Thailand from 2008 to 2009 (*n* = 18,323) (24). Compared to other chronic liver diseases such as hepatitis B and C, MAFLD typically progresses more slowly. However, given the large number of affected individuals, the resulting health damage and disease burden cannot be underestimated.

MAFLD not only affects the liver but also increases cardiovascular disease risk. Exploring novel biomarkers can aid early intervention and diagnosis. The hallmark of MAFLD is lipid metabolism disorder, primarily manifested as elevated TG, and reduced HDL-C levels. The mechanisms underlying MAFLD caused by lipid abnormalities are multifaceted and complex, with IR playing a pivotal role (25, 26). Under normal physiological conditions, plasma free fatty acid (FFA) levels are higher when fasting. After eating, the increased insulin secretion inhibits the activity of hormone-sensitive lipase, resulting in an anti-lipolytic effect that leads to a decrease in plasma FFA. However, patients with T2DM exhibit peripheral IR, which impairs insulin’s ability to inhibit lipid breakdown in adipose tissue. Hepatic *de novo* synthesis of fat, and elevated serum TG levels collectively drive excessive production and secretion of LDL-C in the liver. This leads to elevated fatty acid levels in the liver, triggering hepatocyte dysfunction, inflammation, and oxidative stress, thereby accelerating the progression of MAFLD (27). Furthermore, IR-induced reduction in TG hydrolysis and impaired HDL-C synthesis result in both increased serum TG levels and decreased HDL-C concentrations (28). These atherosclerotic lipid abnormalities heighten the risk of MAFLD development. Thus, monitoring and screening for atherosclerotic lipid biomarkers proves beneficial for primary prevention of MAFLD.

Previous research has established that conventional lipid parameters—HDL-C, LDL-C, TG, and TC—are directly or indirectly associated with MAFLD (29–32). In this study, TG and HDL-C were identified as the most valuable conventional lipid parameters for MAFLD screening (Supplementary Figure 2). These results not only corroborate prior findings but also offer robust evidence supporting the clinical utility of TG and HDL-C as conventional lipid parameters in both the screening and management of MAFLD. In recent years, novel lipid parameters have garnered considerable scientific attention, as multiple studies confirm their substantial association with MAFLD. These emerging markers demonstrate meaningful utility in evaluating the risk of MAFLD. Nevertheless, determining which of these non-conventional lipid parameters holds the greatest clinical relevance for MAFLD detection continues to be an unresolved question.

In this retrospective single-center study, analysis of health examination data from Chinese adults revealed that eight non-conventional lipid parameters—namely NHHR, BMI, AIP, RC, GHR, LCI, TyG, and TyG-BMI—demonstrated effectiveness in distinguishing MAFLD patients, with the exception of CHG. Based on ROC curve analysis, TyG-BMI exhibited the strongest predictive performance, achieving an AUC of 0.81. TyG-BMI integrates TG levels, FBG, and BMI into a single composite parameter, offering a novel, simple, and cost-effective indicator for clinical use. IR is a well-established pathogenic driver of MAFLD (33). Although the HOMA-IR remains the gold standard for diagnosing IR and demonstrates high accuracy in MAFLD detection, its reliance on complex and costly assays limits its feasibility in large-scale epidemiological studies (34, 35). By contrast, the TyG index—calculated from fasting TG and glucose levels—serves as a practical, noninvasive surrogate marker for IR and has been validated in clinical settings (36). The TyG index has proven effective in identifying individuals at risk for T2DM, CVD, and metabolic syndrome (37). Recent studies further support its utility in detecting MAFLD, including among lean individuals (38–40). Incorporating BMI into the TyG index enhances its clinical relevance, as TyG-BMI better captures the influence of adiposity and body composition on metabolic dysregulation. Evidence suggests that TyG-BMI surpasses TyG alone as a marker of IR, offering improved stratification in populations with diverse metabolic phenotypes (41–43).

Given the established links between MAFLD, IR, and dyslipidemia, the TyG-BMI index is regarded as a promising predictive marker for MAFLD (38). However, findings across epidemiological investigations have been inconsistent (44). For instance, a cross-sectional study conducted in the Suzhong region of China reported that BMI, ALT, TG, TyG, TyG-BMI, and AIP all demonstrated strong predictive performance for MAFLD, with AUC values of 0.834, 0.803, 0.809, 0.822, 0.880, and 0.824, respectively (45). Among these, TyG-BMI exhibited the highest clinical diagnostic utility and decision-making benefit. Similarly, other studies have shown that TyG-BMI achieved the highest diagnostic accuracy in U. S. adolescents (AUC = 0.917) and in Japanese adults undergoing health examinations (AUC = 0.837) (46, 47). Additionally, a cross-sectional study of U. S. adults identified a significant nonlinear relationship between TyG-BMI and MAFLD, with a critical threshold near 176.78 (48).

According to the ROC curve results in this study, TyG-BMI has the highest AUC is 0.81. Consistent with previous research, RCS revealed a significant nonlinear relationship between TyG-BMI and MAFLD (*P* for nonlinearity<0.001). Subsequent threshold analysis indicated an inflection point value of about 222.426 for TyG-BMI. On the left side of the threshold value, each unit increase in TyG-BMI was associated with a 5.2% increase in the odds of developing MAFLD (OR = 1.052, 95% CI: 1.04–1.064, *P* < 0.001). The effect size (OR) for the right side of the threshold value was 1.02 (95% CI: 1.009–1.031, *P* < 0.001). Clinically, this threshold can assist physicians in risk stratification for MAFLD, to determine whether more aggressive management measures or closer monitoring are necessary. Therefore, incorporating the TyG-BMI threshold as part of a comprehensive risk assessment tool can aid in the development of public health policies and preventive measures.

Subgroup analyses revealed that TyG-BMI demonstrated stronger predictive performance for MAFLD in females compared to males. In general, females exhibit a lower overall risk of MAFLD than males. The peak incidence of MAFLD in males occurs between 40 and 49 years of age, whereas in females it typically presents around 60–69 years, a delay of approximately two decades that is largely attributed to the protective effects of estrogen. However, following menopause, the prevalence of MAFLD in women approaches that of men, likely due to factors such as weight gain, altered fat distribution, dyslipidemia, and declining estrogen levels—all of which elevate MAFLD risk. Furthermore, studies such as that by Boushehri et al. highlight sex-based differences in how TyG-BMI relates to MAFLD, suggesting that identical increases in TyG-BMI may confer differing risk profiles between sexes (49). The precise biological mechanisms underlying these sex differences in MAFLD pathogenesis and presentation remain incompletely understood but may involve variations in carbohydrate and lipid metabolism, as well as menopause-related changes in adiposity and metabolic susceptibility (50). Subgroup analyses also indicated that TyG-BMI was a more accurate predictor in non-diabetic individuals compared to those with diabetes. A systematic review and meta-analysis confirmed that TyG-BMI maintains strong diagnostic accuracy for MAFLD, with the highest AUC observed in the general population and lower values in patients with T2DM (50). This finding appears contradictory given that individuals with MAFLD are at elevated risk of developing diabetes; for instance, a biopsy-confirmed retrospective cohort study reported diabetes incidence rates of 51% in MAFLD patients with advanced fibrosis (F3–F4) and 31% in those with mild-to-moderate fibrosis (F0–F2) (51). One plausible explanation is that the presence of diabetes may attenuate the discriminative performance of TyG-BMI, possibly due to more pronounced and complex metabolic dysregulation in this population. Nevertheless, these observations warrant further validation in larger prospective cohort studies.

Moreover, studies indicate that CVD represents the most common and clinically significant extrahepatic manifestation in individuals with MAFLD, serving as the primary cause of mortality and accounting for approximately 40–45% of all deaths in this population. Extrahepatic malignancies—such as endometrial, breast, prostate, colorectal, and lung cancers—along with liver-related complications constitute the subsequent leading causes (52, 53). Consequently, we extended our analysis to examine the relationship between TyG-BMI and ASCVD risk. Our findings demonstrate a significant association, with MAFLD patients in the highest TyG-BMI tertile exhibiting a markedly elevated ASCVD risk compared to those in the lowest tertile.

MAFLD and CVD are closely linked in their pathophysiological mechanisms, with shared risk factors including IR, inflammatory responses, endothelial cell damage, and lipid accumulation. At the metabolic level, chronic hyperinsulinemia caused by IR accelerates the progression of atherosclerosis by activating vascular smooth muscle cell proliferation and promoting vascular wall inflammation. At the inflammatory level, systemic low-grade inflammation induced by hepatic steatosis triggers endothelial dysfunction and increased plaque instability through the TNF-*α*/NF-κB signaling pathway. At the lipid metabolism level, abnormal secretion of hepatic very low-density lipoprotein (VLDL) leads to the formation of atherosclerotic lipoprotein phenotype, characterized by increased small and dense LDL-C and impaired HDL-C functionality. In individuals diagnosed with T2DM and a BMI greater than 23 kg/m^2^, the presence of MAFLD is positively correlated with an increased risk of CVD. Clinical studies have demonstrated that patients with elevated liver triglyceride levels exhibit reduced myocardial perfusion, decreased glucose uptake in the myocardium, and impaired myocardial energy metabolism, regardless of myocardial ischemia presence. Notably, no significant changes were observed in cardiac mass or functional parameters. These findings highlight the substantial predictive value of liver triglyceride levels for myocardial metabolic impairment and coronary artery dysfunction (54). Therefore, TyG-BMI which combines TG levels, glucose levels, and BMI can effectively reflect the CVD risk in patients with MAFLD. Liver fibrosis is a significant component of the MAFLD. Regardless of BMI, the presence of two or more metabolic disorders is significantly associated with an increased risk of liver fibrosis in MAFLD patients. However, there was no significant association between TyG-BMI and FIB-4 in participants with MAFLD in our study. Nevertheless, the OR in the highest tertile was 1.8, which was greater than 1, potentially indicating a risk, and further relevant studies were still required. Some degree of population selection bias may led to this results. FIB-4 is a non-invasive index and cannot fully meet the criteria for pathological diagnosis. TyG-BMI primarily reflects early metabolic disturbances, whereas FIB-4 assesses structural fibrosis damage. This discovery suggests that risk stratification for MAFLD requires the integration of multidimensional biomarkers, as reliance solely on metabolic parameters may underestimate fibrosis risk.

This study represents a comprehensive assessment comparing the strength of association and diagnostic accuracy of nine non-conventional lipid parameters for MAFLD. Our findings indicate that these novel lipid parameters, particularly TyG-BMI, demonstrate superior identification capability for MAFLD compared to conventional lipid parameters. Furthermore, we established optimal cutoff values and corresponding AUCs for TyG-BMI in MAFLD detection. Through multiple sensitivity analyses, the robustness of these associations was confirmed. Exploratory stratified analyses also yielded insights supporting precision medicine approaches. Finally, TyG-BMI was identified as an independent predictor of ASCVD risk in MAFLD patients, highlighting its potential clinical utility in early cardiovascular risk stratification within this population.

Despite these findings, our study has several limitations. First, given its cross-sectional design, no causal inference can be drawn regarding the relationship between TyG-BMI and MAFLD. Although TyG-BMI demonstrated superior predictive ability for MAFLD risk compared with other parameters, its clinical utility requires further validation in prospective studies. Second, as this is a retrospective study, the analysis was limited by incomplete collection of metabolic risk-related clinical data—such as hs-CRP, WC, and HOMA-IR—which may have contributed to under-diagnosis of MAFLD in some individuals. This have led to some degree of selection bias into the study, as less healthy older adults—who often carry higher CVD risk—are generally less likely to participate in medical health examination. Consequently, our study may represent a healthier subset of the target population, potentially resulting in an underestimation of the true association strength. Third, MAFLD diagnosis was based on ultrasonography rather than liver biopsy, which, while non-invasive and suitable for large-scale screening, has lower accuracy than histopathological examination. Fourth, although multiple covariates were adjusted for, residual confounding from factors such as family history of MAFLD, genetic susceptibility to cholesterol metabolism abnormalities, and other heritable influences cannot be ruled out. Fifth, this study utilized MAFLD criteria reflecting the diagnostic framework prevalent during our data collection period (2020–2021). While MASLD nomenclature has since been adopted, the core diagnostic requirements—hepatic steatosis with metabolic risk factors—remain substantially unchanged. Therefore, we expect the observed performance of lipid parameters to remain broadly informative for MASLD-related hepatic steatosis; however, the change in criteria may introduce some degree of misclassification and could modestly affect cross-study comparability and the direct generalizability of specific estimates. Finally, as a single-center retrospective study conducted in Chinese adults, the generalizability of our results to other populations requires further investigation from external validation cohorts in other centers and contexts.

## 5 Conclusion

This study demonstrates that among the nine evaluated non-conventional lipid parameters, all except CHG—namely BMI, NHHR, AIP, RC, GHR, LCI, TyG, and TyG-BMI—showed positive associations with MAFLD risk. Among these, TyG-BMI exhibited the strongest predictive performance for MAFLD in the Chinese adult population and also served as an independent predictor of ASCVD risk in patients with MAFLD. As a non-invasive and easily accessible parameter, TyG-BMI holds promise as a practical and reliable tool in clinical practice for MAFLD assessment, offering a feasible means for early identification and risk stratification.

## Data Availability

Publicly available datasets were analyzed in this study. This data can be found at: https://datadryad.org/dataset/doi:10.5061/dryad.7d7wm3809.

## References

[1] Eslam M Sarin SK Wong VW Fan JG Kawaguchi T Ahn SH . The Asian Pacific Association for the Study of the liver clinical practice guidelines for the diagnosis and management of metabolic associated fatty liver disease. Hepatol Int. (2020) 14:889–919. doi: 10.1007/s12072-020-10094-2, 33006093

[2] Eslam M Fan JG Yu ML Wong VW Cua IH Liu CJ . The Asian Pacific association for the study of the liver clinical practice guidelines for the diagnosis and management of metabolic dysfunction-associated fatty liver disease. Hepatol Int. (2025) 19:261–301. doi: 10.1007/s12072-024-10774-3 40016576

[3] Theofilis P Vordoni A Kalaitzidis RG. Interplay between metabolic dysfunction-associated fatty liver disease and chronic kidney disease: epidemiology, pathophysiologic mechanisms, and treatment considerations. World J Gastroenterol. (2022) 28:5691–706. doi: 10.3748/wjg.v28.i39.5691, 36338895 PMC9627426

[4] European Association for the Study of the Liver (EASL), European Association for the Study of Diabetes (EASD), European Association for the Study of Obesity (EASO). EASL-EASD-EASO clinical practice guidelines on the management of metabolic dysfunction-associated steatotic liver disease (MASLD). J Hepatol. (2024) 81:492–542. doi: 10.1016/j.jhep.2024.04.031, 38851997

[5] Shimose S Tsutsumi T Nakano D Sano T Amano K Kawaguchi T. An ever-increasing metabolic dysfunction-associated fatty liver disease-related hepatocellular carcinoma: what are problems and countermeasures? Hepatobiliary Surg Nutr. (2023) 12:941–4. doi: 10.21037/hbsn-23-538, 38115921 PMC10727816

[6] Rinella ME Neuschwander-Tetri BA Siddiqui MS Abdelmalek MF Caldwell S Barb D . AASLD practice guidance on the clinical assessment and management of nonalcoholic fatty liver disease. Hepatology. (2023) 77:1797–835. doi: 10.1097/HEP.0000000000000323 36727674 PMC10735173

[7] Kanwal F Shubrook JH Younossi Z Natarajan Y Bugianesi E Rinella ME . Preparing for the NASH epidemic: a call to action. Gastroenterology. (2021) 161:1030–1042.e8. doi: 10.1053/j.gastro.2021.04.074 34416976

[8] Zhong H Dong J Zhu L Mao J Zhao Y Zou Y . Non-alcoholic fatty liver disease: pathogenesis and models. Am J Transl Res. (2024) 16:387–99. doi: 10.62347/KMSA5983, 38463579 PMC10918142

[9] Kwok R Choi KC Wong GL Zhang Y Chan HL Luk AO . Screening diabetic patients for non-alcoholic fatty liver disease with controlled attenuation parameter and liver stiffness measurements: a prospective cohort study. Gut. (2016) 65:1359–68. doi: 10.1136/gutjnl-2015-309265, 25873639

[10] Xing Y Chen J Liu J Ma H. Associations between GGT/HDL and MAFLD: a cross-sectional study. Diabetes Metab Syndr Obes. (2022) 15:383–94. doi: 10.2147/DMSO.S342505, 35177915 PMC8843704

[11] Ma XM Guo YM Jiang SY Li K-X Zheng Y-F Guo X-G . Potential predictive role of non-HDL to HDL cholesterol ratio (NHHR) in MASLD: focus on obese and type 2 diabetic populations. BMC Gastroenterol. (2025) 25:79. doi: 10.1186/s12876-025-03659-8, 39948471 PMC11827471

[12] Chen Y Lu C Ju H Zhou Q Zhao X. Elevated AIP is associated with the prevalence of MAFLD in the US adults: evidence from NHANES 2017-2018. Front Endocrinol (Lausanne). (2024) 15:1405828. doi: 10.3389/fendo.2024.1405828, 38808115 PMC11130487

[13] Liu J Zhou L An Y Wang Y Wang G. The atherogenic index of plasma: a novel factor more closely related to non-alcoholic fatty liver disease than other lipid parameters in adults. Front Nutr. (2022) 9:954219. doi: 10.3389/fnut.2022.954219, 36118762 PMC9478109

[14] Li X Li ZF Wu NQ. Remnant cholesterol and residual risk of atherosclerotic cardiovascular disease. Rev Cardiovasc Med. (2025) 26:25985. doi: 10.31083/RCM25985, 40026498 PMC11868899

[15] Mo D Zhang P Zhang M Dai H Guan J. Cholesterol, high-density lipoprotein, and glucose index versus triglyceride-glucose index in predicting cardiovascular disease risk: a cohort study. Cardiovasc Diabetol. (2025) 24:116. doi: 10.1186/s12933-025-02675-y, 40065297 PMC11895360

[16] Jin X Xu J Weng X. Correction: correlation between ratio of fasting blood glucose to high density lipoprotein cholesterol in serum and non-alcoholic fatty liver disease in American adults: a population based analysis. Front Med (Lausanne). (2025) 12:1683822. doi: 10.3389/fmed.2025.1683822, 40917861 PMC12412186

[17] Qiu X Han Y Cao C Liao Y Hu H. Association between atherogenicity indices and prediabetes: a 5-year retrospective cohort study in a general Chinese physical examination population. Cardiovasc Diabetol. (2025) 24:220. doi: 10.1186/s12933-025-02768-8, 40399916 PMC12096774

[18] Sedibe A Maswanganyi K Mzimela NC Gamede M. Prevalence of metabolic dysfunction-associated steatotic liver disease in people living with HIV and on antiretroviral treatment: a systematic review and meta-analysis protocol. Health Sci Rep. (2024) 7:e70071. doi: 10.1002/hsr2.70071, 39474343 PMC11518884

[19] Hagström H Shang Y Hegmar H Nasr P. Natural history and progression of metabolic dysfunction-associated steatotic liver disease. Lancet Gastroenterol Hepatol. (2024) 9:944–56. doi: 10.1016/S2468-1253(24)00193-6, 39243773

[20] Yan F Nie G Zhou N Zhang M Peng W. Association of fat-to-muscle ratio with non-alcoholic fatty liver disease: a single-Centre retrospective study. BMJ Open. (2023) 13:e072489. doi: 10.1136/bmjopen-2023-072489, 37903611 PMC10618979

[21] Yan Fengqin Nie Guqiao Zhou Nianli Zhang Meng Peng Wen (2023). Association of fat-to-muscle ratio with non-alcoholic fatty liver disease: A single-Centre retrospective study. Dryad. Available online at: 10.5061/dryad.7d7wm3809 (Accessed Sep 1, 2025). PMC10618979 37903611

[22] Chang M Shao Z Shen G. Association between triglyceride glucose-related markers and the risk of metabolic-associated fatty liver disease: a cross- sectional study in healthy Chinese participants. BMJ Open. (2023) 13:e 070189. doi: 10.1136/bmjopen-2022-070189, 37130686 PMC10163481

[23] Ma X Jia J Cui H Zhou J Tian F Yang J . Association between the triglyceride to high density lipoprotein cholesterol ratio and the incidence of metabolic dysfunction- associated fatty liver disease: a retrospective cohort study. BMC Gastroenterol. (2024) 24:389. doi: 10.1186/s12876-024-03471-w, 39487389 PMC11528993

[24] Charatcharoenwitthaya P Karaketklang K Aekplakorn W. Impact of metabolic phenotype and alcohol consumption on mortality risk in metabolic dysfunction-associated fatty liver disease: a population-based cohort study. Sci Rep. (2024) 14:12663. doi: 10.1038/s41598-024-63453-6, 38830939 PMC11148152

[25] Bungau S Behl T Tit DM Tit D Banica F Bratu O . Interactions between leptin and insulin resistance in patients with prediabetes, with and without NAFLD. Exp Ther Med. (2020) 20:197. doi: 10.3892/etm.2020.9327, 33123227 PMC7588790

[26] Barber TM Kabisch S Pfeiffer AFH Weickert MO. Metabolic-associated fatty liver disease and insulin resistance: a review of complex interlinks. Meta. (2023) 13:757. doi: 10.3390/metabo13060757, 37367914 PMC10304744

[27] Ahmed ES Mohamed HE Farrag MA. Luteolin loaded on zinc oxide nanoparticles ameliorates non-alcoholic fatty liver disease associated with insulin resistance in diabetic rats via regulation of PI3K/AKT/FoxO1 pathway. Int J Immunopathol Pharmacol. (2022) 36:3946320221137435. doi: 10.1177/03946320221137435, 36319192 PMC9630902

[28] Zhou Y Yang G Qu C Chen J Qian Y Yuan L . Predictive performance of lipid parameters in identifying undiagnosed diabetes and prediabetes: a cross-sectional study in eastern China. BMC Endocr Disord. (2022) 22:76. doi: 10.1186/s12902-022-00984-x, 35331213 PMC8952267

[29] Wu T Ye J Shao C Li F Lin Y Ma Q . Varied relationship of lipid and lipoprotein profiles to liver fat content in phenotypes of metabolic associated fatty liver disease. Front Endocrinol (Lausanne). (2021) 12:691556. doi: 10.3389/fendo.2021.691556, 34899591 PMC8662313

[30] Guan L Zhang X Tian H Jin X Fan H Wang N . Prevalence and risk factors of metabolic-associated fatty liver disease during 2014-2018 from three cities of Liaoning Province: an epidemiological survey. BMJ Open. (2022) 12:e047588. doi: 10.1136/bmjopen-2020-047588, 35177440 PMC8860048

[31] Hatanaka T Kakizaki S Saito N Nakano Y Nakano S Hazama Y . Impact of Pemafibrate in patients with hypertriglyceridemia and metabolic dysfunction-associated fatty liver disease pathologically diagnosed with non-alcoholic steatohepatitis: a retrospective, single-arm study. Intern Med. (2021) 60:2167–74. doi: 10.2169/internalmedicine.6574-20, 33612679 PMC8355409

[32] Liao X Ma Q Wu T Shao C Lin Y Sun Y . Lipid-lowering responses to Dyslipidemia determine the efficacy on liver enzymes in metabolic dysfunction- associated fatty liver disease with hepatic injuries: a prospective cohort study. Diabetes Metab Syndr Obes. (2022) 15:1173–84. doi: 10.2147/DMSO.S356371, 35464261 PMC9030404

[33] Yu R Xie W Peng H Lu L Yin S Xu S . Diagnostic value of triglyceride-glucose index and related parameters in metabolism-associated fatty liver disease in a Chinese population: a cross-sectional study. BMJ Open. (2023) 13:e075413. doi: 10.1136/bmjopen-2023-07541 PMC10546149 37775293

[34] Han M Wang H Yang S Zhu S Zhao G Shi H . Triglyceride glucose index and atherogenic index of plasma for predicting colorectal neoplasms in patients without cardiovascular diseases. Front Oncol. (2022) 12:1031259. doi: 10.3389/fonc.2022.1031259, 36452491 PMC9702061

[35] Luo E Wang D Yan G Qiao Y Liu B Hou J . High triglyceride-glucose index is associated with poor prognosis in patients with acute ST-elevation myocardial infarction after percutaneous coronary intervention. Cardiovasc Diabetol. (2019) 18:150. doi: 10.1186/s12933-019-0957-3, 31722708 PMC6852896

[36] Alizargar J Bai CH Hsieh NC Wu SV. Use of the triglyceride-glucose index (TyG) in cardiovascular disease patients. Cardiovasc Diabetol. (2020) 19:8. doi: 10.1186/s12933-019-0982-2 31941513 PMC6963998

[37] Sun Y Ji H Sun W An X Lian F. Triglyceride glucose (TyG) index: a promising biomarker for diagnosis and treatment of different diseases. Eur J Intern Med. (2025) 131:3–14. doi: 10.1016/j.ejim.2024.08.026, 39510865

[38] Zhang R Guan Q Zhang M Ding Y Tang Z Wang H . Association between triglyceride-glucose index and risk of metabolic dysfunction-associated fatty liver disease: a cohort study. Diabetes Metab Syndr Obes. (2022) 15:3167–79. doi: 10.2147/DMSO.S383907, 36268197 PMC9578360

[39] Ying Y Ji Y Ju R Chen J Chen M. Association between the triglyceride-glucose index and liver fibrosis in adults with metabolism-related fatty liver disease in the United States: a cross-sectional study of NHANES 2017-2020. BMC Gastroenterol. (2025) 25:3. doi: 10.1186/s12876-024-03579-z 39748306 PMC11697960

[40] Zhu R Xu C Jiang S Xia J Wu B Zhang S . Risk factor analysis and predictive model construction of lean MAFLD: a cross-sectional study of a health check-up population in China. Eur J Med Res. (2025) 30:137. doi: 10.1186/s40001-025-02373-1, 40001266 PMC11863909

[41] Lim J Kim J Koo SH Kwon GC. Comparison of triglyceride glucose index, and related parameters to predict insulin resistance in Korean adults: An analysis of the 2007-2010 Korean National Health and nutrition examination survey. PLoS One. (2019) 14:e0212963. doi: 10.1371/journal.pone.0212963, 30845237 PMC6405083

[42] Rao X Xin Z Yu Q Feng L Shi Y Tang T . Triglyceride-glucose-body mass index and the incidence of cardiovascular diseases: a meta-analysis of cohort studies. Cardiovasc Diabetol. (2025) 24:34. doi: 10.1186/s12933-025-02584-0, 39844258 PMC11756031

[43] Hu M Yang J Gao B Wu Z Wu Y Hu D . Prediction of MASLD using different screening indexes in Chinese type 2 diabetes mellitus. Diabetol Metab Syndr. (2025) 17:10. doi: 10.1186/s13098-024-01571-x, 39780236 PMC11716454

[44] Wang J Yan S Cui Y Chen F Piao M Cui W. The diagnostic and prognostic value of the triglyceride-glucose index in metabolic dysfunction-associated fatty liver disease (MAFLD): a systematic review and meta-analysis. Nutrients. (2022) 14:4969. doi: 10.3390/nu14234969, 36500999 PMC9741077

[45] Zhang S Liang H Liu J Zhu Y. Analysis of risk factors associated with metabolic-associated fatty liver disease from Suzhong region, China: a cross-sectional study. BMJ Open. (2025) 15:e096598. doi: 10.1136/bmjopen-2024-096598, 41146374 PMC12557766

[46] Zou Y Dai Y Li Z Lin B Chen H Zhuang Z . Modified triglyceride-glucose indices as novel predictors of metabolic dysfunction-associated fatty liver disease in US adolescents: a nationally representative study from NHANES 2017-2020. BMC Gastroenterol. (2025) 25:325. doi: 10.1186/s12876-025-03915-x, 40312305 PMC12044992

[47] Otsubo N Fukuda T Cho G Ishibashi F Yamada T Monzen K. Utility of indices obtained during medical checkups for predicting fatty liver disease in non-obese people. Intern Med. (2023) 62:2307–19. doi: 10.2169/internalmedicine.1097-22, 36517035 PMC10484762

[48] Wang Q Su Y Niu J Wang Y Liu L Hao Y. Nonlinear association between TyG-BMI and MAFLD: a cross-sectional study. PLoS One. (2025) 20:e0331140. doi: 10.1371/journal.pone.0331140, 41100462 PMC12530604

[49] Boushehri YG Meymanatabadi Z Tanha AE Azami P Alaei M Alamdari AA . Association of triglyceride glucose-body mass index (TyG-BMI) with metabolic dysfunction-associated steatotic liver disease: a systematic review and meta-analysis. PLoS One. (2025) 20:e0324483. doi: 10.1371/journal.pone.0324483, 40758732 PMC12321072

[50] Ling Q Chen J Liu X Xu Y Ma J Yu P . The triglyceride and glucose index and risk of nonalcoholic fatty liver disease: a dose-response meta-analysis. Front Endocrinol (Lausanne). (2023) 13:1043169. doi: 10.3389/fendo.2022.1043169, 36743937 PMC9892833

[51] Björkström K Stål P Hultcrantz R Hagström H. Histologic scores for fat and fibrosis associate with development of type 2 diabetes in patients with nonalcoholic fatty liver disease. Clin Gastroenterol Hepatol. (2017) 15:1461–8. doi: 10.1016/j.cgh.2017.04.040, 28479500

[52] Lee H Lee YH Kim SU Kim HC. Metabolic dysfunction-associated fatty liver disease and incident cardiovascular disease risk: a nationwide cohort study. Clin Gastroenterol Hepatol. (2021) 19:2138–2147.e10. doi: 10.1016/j.cgh.2020.12.022, 33348045

[53] Thomas JA Kendall BJ Dalais C Macdonald GA Thrift AP. Hepatocellular and extrahepatic cancers in non-alcoholic fatty liver disease: a systematic review and meta-analysis. Eur J Cancer. (2022) 173:250–62. doi: 10.1016/j.ejca.2022.06.051, 35944373

[54] Tian H Zhao X Zhang Y Xia Z. Abnormalities of glucose and lipid metabolism in myocardial ischemia-reperfusion injury. Biomed Pharmacother. (2023) 163:114827. doi: 10.1016/j.biopha.2023.114827, 37141734

