# Supplementary material for: The development and evaluation of nine non-conventional lipid parameters for metabolic dysfunction-associated fatty liver disease in Chinese medical health examination adults: a single-center retrospective study
Source: Front Nutr. 2026 Feb 25;13:1788704. doi: 10.3389/fnut.2026.1788704 (PMC12976015; doi:10.3389/fnut.2026.1788704)
Supplement: Supplementary file 1 [file Data_Sheet_1.docx]

Supplementary Material

# Supplementary Tables

**1.1** **Supplementary Table 1.** The Variance Inflation Factor values of all covariates.

| Variables | Variance Inflation Factor |
| --- | --- |
| Sex | 1.669 |
| Age | 1.304 |
| PLT | 1.129 |
| ALT | 1.93 |
| AST | 1.893 |
| UA | 1.376 |
| FBG | 1.96 |
| TC | 13.747 |
| TG | 6.581 |
| HDL-C | 2.182 |
| LDL-C | 11.065 |
| SBP | 1.722 |
| DBP | 1.613 |
| Tobacco use | 1.555 |
| Alcohol use | 1.559 |
| Hypertension | 1.391 |
| Diabetes | 1.393 |
| BMI | 9.727 |

**1.2 Supplementary Table 2.** Baseline characteristics of individuals with MASLD and MAFLD.

| **Variables** | **MASLD**  **(n = 973)** | **MAFLD**  **(n = 937)** |
| --- | --- | --- |
| Sex, n (%) |  |  |
| Female | 215 (22.1) | 197 (21) |
| Male | 758 (77.9) | 740 (79) |
| Age (years), n (%) |  |  |
| < 60 | 669 (68.8) | 640 (68.3) |
| ≥ 60 | 304 (31.2) | 297 (31.7) |
| PLT (10^9^ /L) | 212.1 ± 53.3 | 212.1 ± 53.8 |
| ALT (U/L) | 29.86±22.0 | 30.2 ± 22.3 |
| AST (U/L) | 24.18±11.5 | 24.3 ± 11.7 |
| UA (umol/L) | 385.17±96.9 | 388.0 ± 96.7 |
| FBG (mmol/L) | 5.77±1.7 | 5.8 ± 1.8 |
| TC (mmol/L) | 4.55±1.1 | 4.5 ± 1.1 |
| TG (mmol/L) | 2.24±1.9 | 2.3 ± 1.9 |
| HDL-C (mmol/L) | 1.05±0.3 | 1.0 ± 0.3 |
| LDL-C (mmol/L) | 2.69±0.9 | 2.7 ± 0.9 |
| SBP (mm Hg) | 132.22±15.9 | 132.6 ± 15.9 |
| DBP (mm Hg) | 82.79±11.1 | 83.1 ± 11.1 |
| Tobacco use, n (%) |  |  |
| NO | 596 (61.3) | 571 (60.9) |
| Yes | 377 (38.7) | 366 (39.1) |
| Alcohol use, n (%) |  |  |
| NO | 616 (63.3) | 588 (62.8) |
| Yes | 357 (36.7) | 349 (37.2) |
| Hypertension, n (%) |  |  |
| NO | 333 (34.2) | 304 (32.4) |
| Yes | 640 (65.8) | 633 (67.6) |
| Diabetes, n (%) |  |  |
| NO | 601 (61.8) | 565 (60.3) |
| Yes | 372 (38.2) | 372 (39.7) |
| BMI (kg/m^2^) | 26.3 ± 2.9 | 26.5 ± 2.8 |
| TyG | 9.0 ± 0.7 | 9.0 ± 0.7 |
| AIP | 0.6 (0.4, 0.8) | 0.6 ± 0.3 |
| GHR | 2.7 ± 1.1 | 2.8 ± 1.1 |
| TyG-BMI | 236.9 ± 35.1 | 238.9 ± 34.2 |
| CHG | 13.4 ± 0.5 | 13.4 ± 0.5 |
| LCI | 20.8 (10.3, 38.9) | 31.7 ± 35.0 |
| NHHR, M (P_25_, P_75_) | 3.5 ± 1.5 | 3.4 (2.5, 4.4) |
| RC, M (P_25_, P_75_) | 0.7 (0.4, 0.9) | 0.7 (0.4, 0.9) |
| FIB-4 score, M (P_25_, P_75_) | 1.2 (0.9, 1.6) | 1.2 (0.9, 1.6) |
| ASCVD risk score, M (P_25_, P_75_) | 10.8 (5.2, 19.6) | 11.0 (5.4, 20.3) |

**Abbreviations:** PLT, platelets; ALT, alanine aminotransferase; AST, aspartate aminotransferase; UA, uric acid; FBG, fasting blood glucose; TC, total cholesterol; TG, triglycerides; HDL-C, high-density lipoprotein cholesterol; LDL-C, low-density lipoprotein cholesterol; SBP, systolic blood pressure; DBP, diastolic blood pressure; BMI, body mass index; TyG, triglyceride-glucose index; AIP, atherogenic Index of Plasma; GHR, fasting blood glucose to HDL-C; LCI, lipoprotein combine index; RC, remnant cholesterol; NHHR, non-HDL-to-HDL cholesterol ratio; CHG, cholesterol, high-density lipoprotein, and glucose index; M (P_25_, P_75_), median (lower quartile, upper quartile); FIB-4 score, fibrosis-4 score; ASCVD, atherosclerotic cardiovascular disease; MASLD, metabolic dysfunction-associated steatotic liver disease.

**1.3 Supplementary Table 3.** Association between TyG-BMI and MASLD or MAFLD in different models.

| **Criteria** | **Variables** | **Model 1** |  | **Model 2** |  | **Model 3** |  |
| --- | --- | --- | --- | --- | --- | --- | --- |
|  |  | **OR (95% CI)** | ***P* value** | **OR (95% CI)** | ***P* value** | **OR (95% CI)** | ***P* value** |
| MASLD | TyG-BMI | 1.04(1.03-1.04) | < 0.001 | 1.04(1.03-1.04) | < 0.001 | 1.03 (1.03-1.04) | < 0.001 |
|  | Per 1-SD increment | 3.95 (3.35-4.66) | < 0.001 | 3.62 (3.04-4.31) | < 0.001 | 3.11 (2.59-3.72) | < 0.001 |
|  | TyG-BMI (tertiles) |  |  |  |  |  |  |
|  | Q1 | Reference |  | Reference |  | Reference |  |
|  | Q2 | 3.3 (2.56-4.24) | < 0.001 | 2.94 (2.27-3.81) | < 0.001 | 2.52 (1.93-3.3) | < 0.001 |
|  | Q3 | 13.73 (10.04-18.78) | < 0.001 | 10.67 (7.69-14.81) | < 0.001 | 7.84 (5.57-11.04) | < 0.001 |
|  | P for trend | < 0.001 | | < 0.001 | | < 0.001 | |
|  |  |  | |  | |  | |
| MAFLD | TyG-BMI | 1.04 (1.04-1.05) | < 0.001 | 1.04 (1.04-1.05) | < 0.001 | 1.04 (1.03-1.04) | < 0.001 |
|  | Per 1-SD increment | 4.85 (4.07-5.79) | < 0.001 | 4.33 (3.6-5.21) | < 0.001 | 3.7 (3.05-4.48) | < 0.001 |
|  | TyG-BMI(tertiles) |  |  |  |  |  |  |
|  | Q1 | Reference |  | Reference |  | Reference |  |
|  | Q2 | 4.17 (3.22-5.41) | < 0.001 | 3.64 (2.79-4.75) | < 0.001 | 3.14 (2.39-4.13) | < 0.001 |
|  | Q3 | 18.09 (13.15-24.89) | < 0.001 | 13.5 (9.68-18.82) | < 0.001 | 9.87 (6.99-13.94) | < 0.001 |
|  | P for trend | < 0.001 | | <0.001 | | <0.001 | |

**Notes:** Model 1: unadjusted.

Model 2: sex, age, tobacco use, alcohol use, hypertension, diabetes.

Model 3: model 2+ ALT, AST, UA, PLT, SBP, DBP.

**Abbreviations:** OR, odds ratio; CI, confidence interval.

**1.4 Supplementary Table 4.** Nonlinearity addressed through two-piecewise linear model.

|  | MAFLD | |
| --- | --- | --- |
|  | OR (95%CI) | *P* value |
| Threshold value | 222.426 |  |
| < Threshold value | 1.052 (1.04-1.064) | < 0.001 |
| > Threshold value | 1.02 (1.009-1.031) | < 0.001 |

**Notes:** The OR was calculated based on a multivariable logistic regression model adjusted for Model 3.

**Abbreviations:** OR, odds ratio.

# 1.5 Supplementary Table 5. Interaction between the TyG-BMI and sex, diabetes on MAFLD.

| Interactive indices | Interactive effects (95% CI) | | |
| --- | --- | --- | --- |
|  | Model 1 | Model 2 | Model 3 |
| *TyG-BMI & Sex* |  |  |  |
| Additive effect |  |  |  |
| RERI | -1.05(-7.52-5.41) | -2.77 (-8.38-2.84) | -3.58 (-7.47-0.32) |
| AP | -0.08 (-0.61-0.44) | -0.33 (-1.02-0.36) | -0.96 (-2.09-0.16) |
| SI | 0.92 (0.54-1.55) | 0.73 (0.41-1.3) | 0.43 (0.21-0.87) |
| Multiplicative effect | 0.52 (0.29-0.94) | 0.51 (0.28-0.93) | 0.53 (0.29-0.99) |
|  |  |  |  |
| *TyG-BMI & Diabetes* |  |  |  |
| Additive effect |  |  |  |
| RERI | 2.58 (-2.03-7.19) | 1.94 (-1.98-5.85) | 1.71 (-1.29-4.7) |
| AP | 0.21 (-0.1-0.52) | 0.19 (-0.14-0.51) | 0.22(-0.1-0.53) |
| SI | 1.29 (0.84-1.98) | 1.26 (0.81-1.95) | 1.33 (0.83-2.13) |
| Multiplicative effect | 0.61 (0.36-1.02) | 0.64 (0.38-1.08) | 0.73 (0.43-1.25) |

**Notes:** Model 1: unadjusted.

Model 2: sex, age, tobacco use, alcohol use, hypertension, diabetes.

Model 3: model 2+ ALT, AST, UA, PLT, SBP, DBP.

**1.6** **Supplementary Table 6.**  Receiver operating characteristic curve analysis of TyG-BMI for the detection of MAFLD.

| Variables | Subgroup | Cut-off value | Sensitivity | Specificity | AUC (95% CI) | *p* value (adjusted) |
| --- | --- | --- | --- | --- | --- | --- |
| Sex | Female | 202.04 | 0.83 | 0.73 | 0.86(0.83, 0.89) | < 0.001 |
|  | Male | 219.31 | 0.72 | 0.72 | 0.78(0.75, 0.81) | < 0.001 |
| Diabetes | No | 216.98 | 0.68 | 0.81 | 0.83(0.8, 0.85) | < 0.001 |
|  | Yes | 221.19 | 0.74 | 0.66 | 0.74(0.69, 0.79) | < 0.001 |

**1.7** **Supplementary Table 7.**  Association between TyG-BMI and the risk of ASCVD and liver fibrosis in patients with MASLD or MAFLD.

| Criteria | Outcome  variables | Variables | Model 1 |  | Model 2 |  | Model 3 |  |
| --- | --- | --- | --- | --- | --- | --- | --- | --- |
|  |  |  | **OR (95% CI)** | **P value** | **OR (95% CI)** | **P value** | **OR (95% CI)** | **P value** |
| MASLD | ASCVD | TyG-BMI | 1.01(1.01-1.01) | <0.001 | 1.01 (1-1.01) | 0.007 | 1.01 (1-1.01) | 0.052 |
|  |  | TyG-BMI(tertiles) | | | | | | |
|  |  | Q1 | Reference |  | Reference |  | Reference |  |
|  |  | Q2 | 1.55 (1.07-2.25) | 0.021 | 1.91 (1.04-3.53) | 0.038 | 1.85 (0.97-3.5) | 0.061 |
|  |  | Q3 | 2.72 (1.91-3.89) | <0.001 | 3.03(1.67-5.48) | <0.001 | 2.56 (1.37-4.8) | 0.003 |
|  |  | P for trend | < 0.001 | | < 0.001 | | 0.004 | |
|  | Liver fibrosis risk | TyG-BMI | 1 (0.99-1) | 0.371 | 1 (1-1.01) | 0.69 | 1 (0.99-1.01) | 0.946 |
|  |  | TyG-BMI(tertiles) | | | | | | |
|  |  | Q1 | Reference |  | Reference |  | Reference |  |
|  |  | Q2 | 1.03 (0.71-1.49) | 0.882 | 1.03 (0.67-1.58) | 0.9 | 1.29 (0.61-2.74) | 0.51 |
|  |  | Q3 | 0.97 (0.68-1.38) | 0.87 | 1.13 (0.74-1.72) | 0.569 | 1.41 (0.65-3.05) | 0.389 |
|  |  | P for trend | 0.797 | | 0.517 | | 0.408 | |
|  |  |  |  | |  | |  | |
| MAFLD | ASCVD | TyG-BMI | 1.01(1.01-1.01) | <0.001 | 1.01 (1-1.01) | 0.016 | 1.01 (1-1.01) | 0.087 |
|  |  | TyG-BMI(tertiles) | | | | | | |
|  |  | Q1 | Reference |  | Reference |  | Reference |  |
|  |  | Q2 | 1.35 (0.91-2.01) | 0.13 | 1.97 (1.04-3.73) | 0.039 | 1.89 (0.97-3.69) | 0.063 |
|  |  | Q3 | 2.31 (1.58-3.36) | <0.001 | 3.01 (1.62-5.59) | <0.001 | 2.55 (1.33-4.91) | 0.005 |
|  |  | P for trend | < 0.001 | | < 0.001 | | 0.006 | |
|  | Liver fibrosis risk | TyG-BMI | 1 (0.99-1) | 0.342 | 1 (1-1.01) | 0.503 | 1 (0.99-1.01) | 0.766 |
|  |  | TyG-BMI(tertiles) | | | | | | |
|  |  | Q1 | Reference |  | Reference |  | Reference |  |
|  |  | Q2 | 1.05 (0.71-1.56) | 0.805 | 1.13 (0.72-1.79) | 0.592 | 1.73 (0.75-3.99) | 0.197 |
|  |  | Q3 | 0.98 (0.67-1.43) | 0.912 | 1.23 (0.79-1.92) | 0.361 | 1.8 (0.78-4.14) | 0.168 |
|  |  | P for trend | 0.792 | | 0.356 | | 0.224 | |

**Notes:** Model 1: unadjusted.

Model 2: sex, age, tobacco use, alcohol use, hypertension, diabetes.

Model 3: model 2+ ALT, AST, UA, PLT, SBP, DBP.

Abbreviations: OR, odds ratio; CI, confidence interval; ASCVD, atherosclerotic cardiovascular disease.

## 2 Supplementary Figures

| A | B | C | D |
| --- | --- | --- | --- |
| 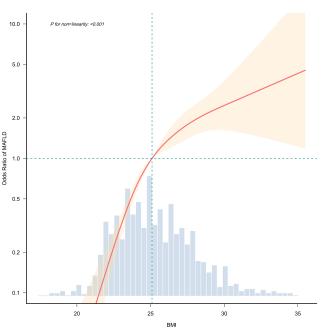 | 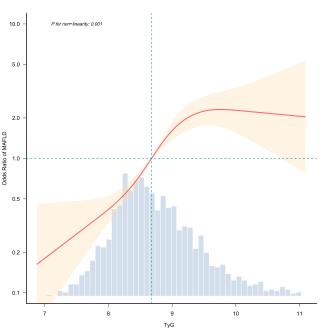 | 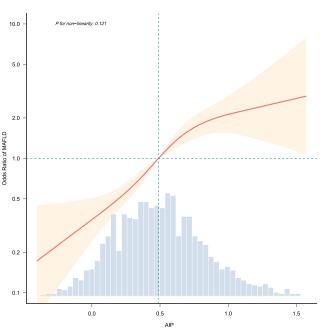 | 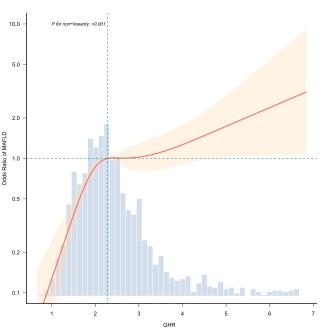 |
| E | F | G | H |
| 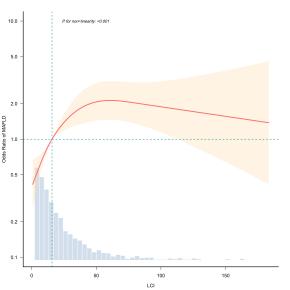 | 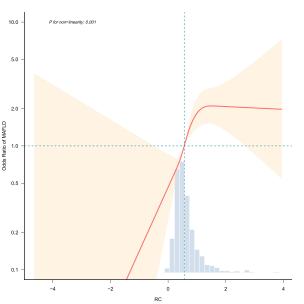 | 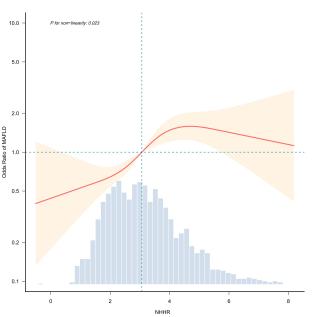 | 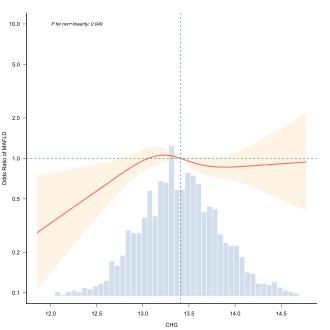 |

**2.1 Supplementary Figure 1.** The RCS analysis of the association between BMI (A), TyG (B), AIP (C), GHR (D), LCI (E), RC (F), NHHR (G), CHG (H), and MAFLD.

| **ROC curves for MAFLD** |
| --- |
| 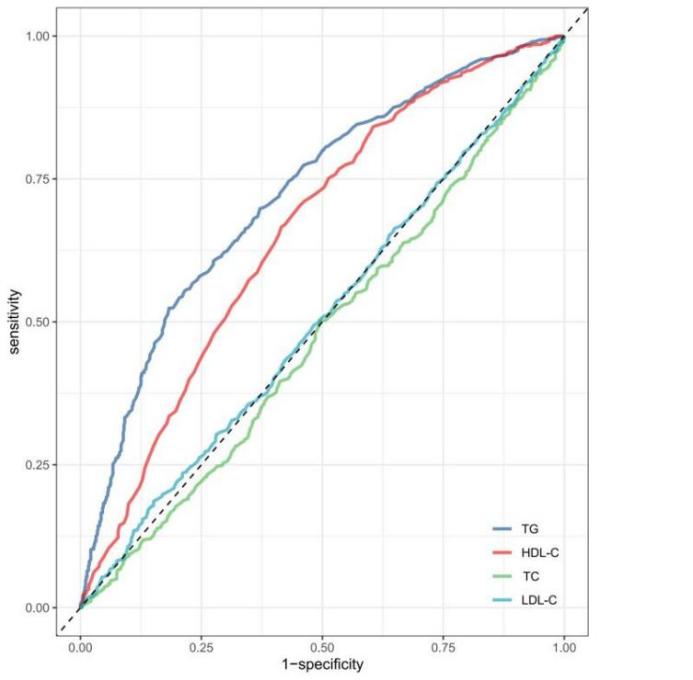 |

**2.2 Supplementary Figure 2.** ROC curves of traditional lipid parameters for MAFLD screening in Chinese adults**.**
